# SiaScoreNet: a siamese neural network-based model integrating prediction scores for HLA-peptide interaction prediction

**DOI:** 10.1093/bioadv/vbaf248

**Published:** 2025-11-19

**Authors:** Mahsa Saadat, Fatemeh Zare-Mirakabad, Milad Besharatifard

**Affiliations:** Computational Biology Research Center (CBRC), Amirkabir University of Technology, Tehran, Iran; Department of Mathematics and Computer Science, Amirkabir University of Technology, Tehran, Iran; Computational Biology Research Center (CBRC), Amirkabir University of Technology, Tehran, Iran; Department of Mathematics and Computer Science, Amirkabir University of Technology, Tehran, Iran; Computational Biology Research Center (CBRC), Amirkabir University of Technology, Tehran, Iran; Department of Mathematics and Computer Science, Amirkabir University of Technology, Tehran, Iran

## Abstract

**Motivation:**

Cancer immunotherapy uses the immune system to recognize and eliminate tumor cells by presenting tumor antigens through Human Leukocyte Antigen (HLA) molecules. Accurate prediction of HLA–peptide interactions is essential for personalized immunotherapy development. Allele-specific models achieve high accuracy and handle variable peptide lengths but require separate training for each allele, limiting scalability to rare or unseen HLAs. Pan-specific models generalize across multiple alleles and match or surpass allele-specific methods. Ensemble methods improve prediction by combining outputs from multiple predictors, often via linear combinations, though nonlinear strategies may better capture HLA–peptide complexities.

We propose *SiaScoreNet*, a three-step predictive pipeline enhancing HLA–peptide interaction prediction. First, ESM, a pretrained transformer-based protein language model, embeds HLA and peptide sequences into fixed-length representations, accommodating varying sequence lengths. Second, we integrate predicted scores from state-of-the-art models into a comprehensive feature vector. Third, a nonlinear ensemble strategy combines features, capturing complex dependencies and boosting performance.

**Results:**

Benchmark evaluations show *SiaScoreNet* outperforms existing models in accuracy, comparable to TransPHLA, BigMHC, and CapHLA. Recent models prioritize recall over precision, valuable for identifying potential binders but resource-intensive. *SiaScoreNet* offers improved performance and runtime efficiency compared to these models, evaluated against HPV viruses for HLA–peptide prediction.

**Availability and implementation:**

The data and source code for prediction and experiments presented in this study is publicly available in the *SiaScoreNet* repository hosted on GitHub: https://github.com/CBRC-lab/SiaScoreNet.

## 1 Introduction

Cancer immunotherapy uses the immune system’s power to combat cancer by targeting tumor antigens presented by Human Leukocyte Antigen (HLA) molecules, also known as the Major Histocompatibility Complex (MHC) in vertebrates. Research indicates that the variety in HLA types may influence susceptibility to certain cancers, such as hepatocellular carcinoma, which is linked to hepatitis B virus infection (Fatima *et al.* 2022; Kawamura *et al.* 2023). Understanding HLA-peptide interactions is crucial for developing personalized immunotherapeutic strategies. Epitopes are specific peptide sequences within antigens that are recognized by T cells. The effectiveness of immunotherapy often depends on the precise identification of these epitopes and their presentation by HLA molecules.

HLA molecules are highly polymorphic proteins encoded by genes on chromosome 6 (Mungall *et al.* 2003). They play an important role in immune surveillance and the activation of T cells against foreign and mutated antigens, including those from tumors (Choo 2007). Specifically, HLA class I proteins (HLA-A, B, and C) present small endogenous peptides to CD8+ cytotoxic T cells, enabling the detection and elimination of infected or malignant cells (Aptsiauri and Garrido 2022). In contrast, HLA class II proteins (HLA-DPB1, DQB1, and DRB1) present larger peptides to CD4+ T cells, initiating adaptive immunity against cancerous cells (Georgopoulos and James 2022). Understanding these distinctions is essential for comprehending their roles in immune responses (Giatromanolaki *et al.* 2024).

Recent findings highlight the significance of HLA-mediated antigen presentation in enabling T cell recognition and destruction of cancer cells, particularly in breast cancer (Luo *et al.* 2015; Giatromanolaki *et al.* 2024). This highlights the dual importance of HLA molecules in both immune response regulation and cancer immunosurveillance. Understanding these mechanisms is pivotal for developing targeted immunotherapies.

Given their role in presenting peptides to T cells, computational methods are indispensable for predicting HLA-peptide interactions, particularly focusing on peptides binding to HLA class I molecules, crucial for immune function (Soria-Guerra *et al.* 2015). Various computational approaches have been developed to predict HLA class I peptide binding, each offering distinct advantages. These approaches can be categorized into scoring function-based and machine learning-based methods.

Scoring function-based methods include the SMM (Peters *et al.* 2003), SMMPMBEC (Kim *et al.* 2009), and PickPocket (Zhang *et al.* 2009). SMM, a matrix-based approach, that predicts MHC-peptide binding affinity by summing the contributions of individual residues. It uses experimental data to create a predictive model that minimizes errors and addresses limitations of small datasets. SMMPMBEC extends SMM by incorporating Bayesian priors, allowing it to better handle limited peptide data and impute missing residue information. PickPocket estimates binding strength by analyzing polymorphic residues in MHC binding pockets, offering structural insights into MHC-peptide interactions.

Machine learning-based methods include various models based on Artificial Neural Networks (ANN) including NetMHC (Lundegaard *et al.* 2008), NetMHCpan (Jurtz *et al.* 2017), and NetMHCStabpan (Rasmussen *et al.* 2016). The NetMHC (Lundegaard *et al.* 2008), has been trained on large-scale peptide to MHC affinity data from databases like IEDB (Vita *et al.* 2019) and SYFPEITHI (Rammensee *et al.* 1999). NetMHCpan (Jurtz *et al.* 2017) is a pan-specific, neural network-based model that provides two types of predictions: binding affinity (BA) and eluted ligand (EL) likelihood. These predictions are generated by two distinct output neurons within the same model. NetMHCpan_BA estimates the binding affinity of peptides to MHC class I allele using a diverse set of affinity measurements, while NetMHCpan_EL predicts the likelihood that a peptide is naturally processed and presented on the cell surface. NetMHCStabpan (Rasmussen *et al.* 2016) predicts the stability of MHC-peptide complexes, which is important for determining immunogenicity. This model combines neural network-based stability predictions with established MHC-peptide binding predictors, improving accuracy in identifying cytotoxic T lymphocyte epitopes compared to traditional affinity-based methods.

In recent years, attention-based deep learning models have shown promising performance in HLA-peptide binding prediction, including CapHLA (Chang and Wu 2024), TransPHLA (Chu *et al.* 2022), and BigMHC (Albert *et al.* 2023). These models demonstrate strong performance but typically require significant computational resources for training. Furthermore, there are approaches that combine multiple prediction models to enhance accuracy. One such approach (Karosiene *et al.* 2012) consolidates the outputs of various methods, including NetMHC (Lundegaard *et al.* 2008), NetMHCpan (Jurtz *et al.* 2017), and PickPocket (Zhang *et al.* 2009), to improve overall performance. Similarly, the Consensus method (Moutaftsi *et al.* 2006) from the Immune Epitope Database Analysis Resource (IEDB-AR (Dhanda *et al.* 2019)) integrates multiple methods, such as NetMHC (Lundegaard *et al.* 2008), SMM (Peters and Sette 2005), and CombLib (Sidney *et al.* 2008), to offer a comprehensive tool for T cell epitope analysis and prediction. This multi-method approach enhances the reliability and robustness of epitope predictions, ensuring a more complete understanding of MHC-peptide interactions. Recently, HLAPepBinder (Saadat *et al.* 2024) has been introduced as an ensemble learning-based model that integrates predictions from nine different HLA class I tools from IEDB-AR (Dhanda *et al.* 2019) to enhance HLA-peptide binding accuracy.

Despite considerable progress in HLA–peptide interaction prediction, several key challenges remain. Early approaches often relied on allele-specific models, which require independent training for each HLA allele, leading to poor scalability and limited generalizability to rare or novel alleles. Pan-specific models were developed to address this issue by learning shared patterns across alleles; a key challenge in their development has been the effective extraction of features from peptides of varying lengths. State-of-the-art methods such as NetMHCpan have addressed this by identifying a central binding motif—typically a 9-mer—using alignment strategies involving insertions and deletions. While this technique is effective at capturing the core binding region, it may overlook sequence information outside the core. Recent transformer-based models have improved feature extraction by encoding sequences into fixed-length embeddings, but they demand substantial computational resources during training time. To reduce computational resource usage, the recent consensus model HLAPepBinder (Saadat *et al.* 2024) combines predictions from IEDB models using a non-linear approach based on random forests. This method achieves performance comparable to transformer-based models while requiring significantly less time and computational resources.

In this study, we explore whether combining the prediction scores from IEDB models with language model representations of HLA and peptide sequences can improve predictive performance. We investigate whether feeding these features into a lightweight neural network with a low parameter count can outperform transformer-based models while remaining computationally efficient.

To explore this hypothesis, we introduce a new pipeline called *SiaScoreNet* which enhances the accuracy of HLA-peptide interaction predictions by utilizing Evolutionary Scale Modeling (ESM) (Lin *et al.* 2023), a transformer-based model pretrained to extract meaningful biological features from both HLA molecules and peptides. Unlike previous models, ESM is flexible, not relying on fixed input sequence lengths, which enables it to capture more nuanced insights from protein sequences (Behjati *et al.* 2022). We then employ a Siamese Neural Network (SNN) to extract the most relevant embedding vectors from the ESM-encoded HLA and peptide representations. Recognizing the complexities in HLA-peptide interactions, our approach integrates insights from existing models and adopts an ensemble strategy to significantly improve predictive performance. By incorporating prediction scores from nine IEDB state-of-the-art models, *SiaScoreNet* offers a comprehensive solution that outperforms established benchmarks in forecasting HLA-peptide interactions. Comparative evaluations demonstrate that *SiaScoreNet* not only outperforms existing computational predictors but also achieves superior runtime efficiency compared to recently proposed transformer-based models. To better show how useful *SiaScoreNet* can be in real situations, we present a case study on Human papillomavirus (HPV). HPV is a common sexually transmitted infection that can lead to several types of cancer, especially cervical cancer. Although there are vaccines to prevent HPV, they are not very effective for treatment (Schiller and Lowy 2012; Trimble *et al.* 2015). This makes it important to accurately find immune-related parts of the virus (called epitopes) to help design better vaccines and treatments.

## 2 Methods

In this section, we define the computational problem of HLA-peptide interaction prediction and provide an overview of the dataset used in our study. We then present the *SiaScoreNet* pipeline, an innovative framework specifically designed to tackle the challenges associated with the HLA-peptide interaction prediction problem.

### 2.1 HLA-peptide interaction prediction problem

**Figure 1.**
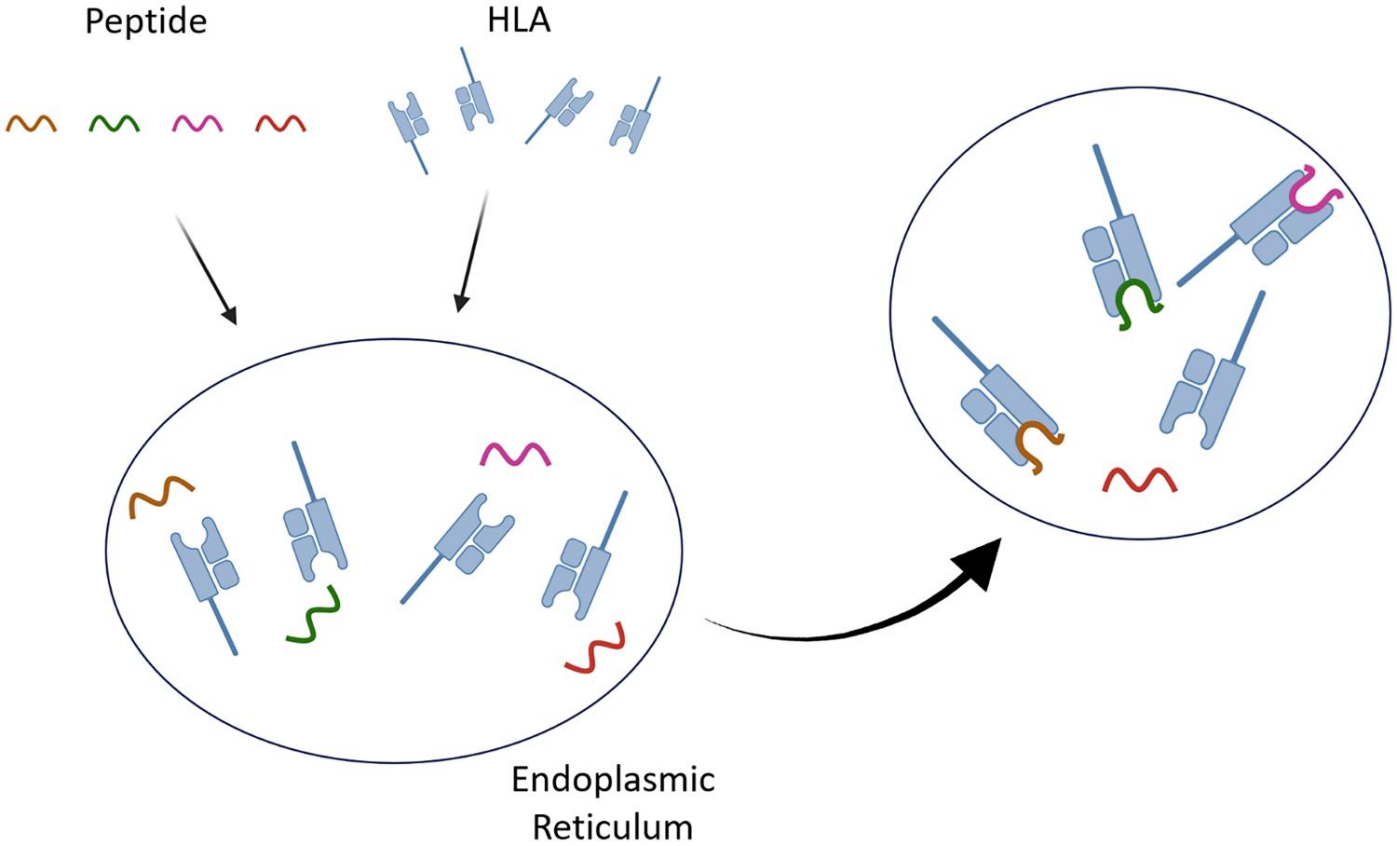
Illustration of the HLA-peptide interaction binary classification problem. HLA class I molecules and peptides are in a shared environment, highlighting potential interactions where peptides may bind to specific HLA molecules or remain unbound.

In Endoplasmic Reticulum (ER), peptides, typically derived from proteasome-degraded proteins, are transported into the ER by the transporter associated with antigen processing. Within the ER, these peptides bind to HLA class I molecules, forming an HLA-peptide complex. These complexes are then transported to the cell surface for presentation to CD8+ cytotoxic T cells, enabling immune surveillance. HLA-peptide interaction prediction problem is formulated as a binary classification problem, where the goal is to determine whether a given peptide interacts with a specific HLA molecule. Figure 1 illustrates this concept by showing HLA class I molecules and peptides placed within ER, where they may or may not form a binding interaction (Klein and Sato 2000).

Here, we define two sets: $H=\{h_{1},\ldots ,h_{n}\}$, consisting of n HLAs, and, $P=\{p_{1},\ldots ,p_{m}\}$, consisting of m peptides. Based on experimental data, we represent the known interactions using an association matrix $Y_{n\times m}$ defined as follows:


yi,j={1,if pj binds to hi,0,if pj does not bind to hi,X,if binding status of pj and hi is unknown. 

The HLA-peptide interaction problem is then defined as:

- Input: HLA $h_{i}\in H$, peptide $p_{j}\in P$ and $y_{i,j}=X$ (unknown interaction).
- Output: Predict $y_{i,j}=1$ or $y_{i,j}=0$, indicating whether the peptide $p_{j}$ is likely to bind to $h_{i}$.

### 2.2 Dataset

Chu *et al.* (Chu *et al.* 2022) used a dataset referred to as the independent test set (denoted as D1) to evaluate their model, TransPHLA, against the predictors provided by IEDB (Dhanda *et al.* 2019). This dataset includes three groups comprising a total of 73 HLA subtypes and 115953 peptides. The details of dataset $D_{1}$ are provided in Table S1. To further evaluate our model, we also utilize an external test set (Chu *et al.* 2022), referred to as $D_{2}$, described in Table S2. This dataset comprises two groups spanning five HLA subtypes: HLA-A*01:01, HLA-A*02:01, HLA-A*24:02, HLA-B*08:01, and HLA-B*18:01. There is no overlap in HLA-peptide pairs between $D_{1}$ and $D_{2}$, ensuring an unbiased evaluation.

### 2.3 SiaScoreNet pipeline

**Figure 2.**
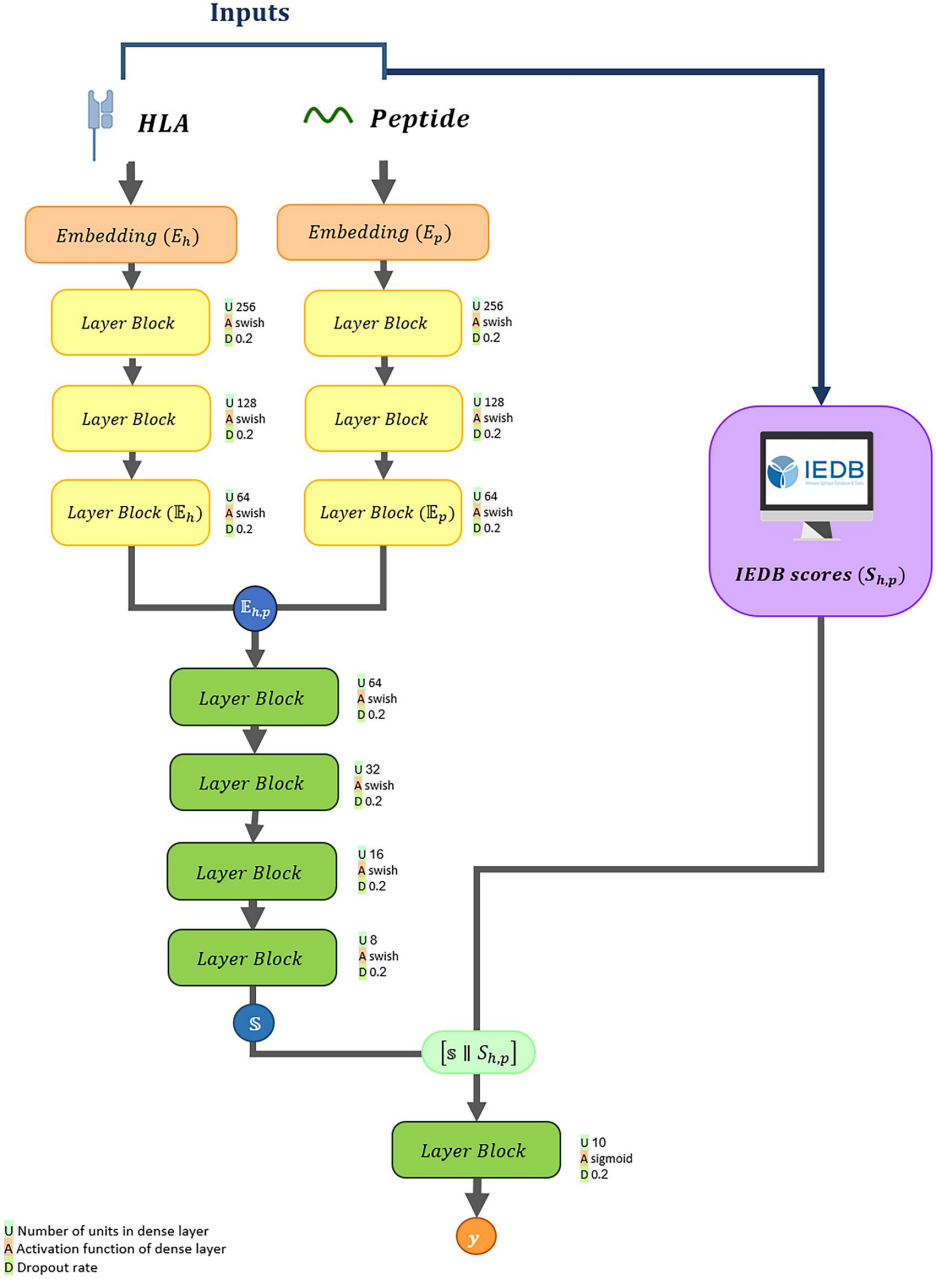
Architecture of the *SiaScoreNet* pipeline. The pipeline takes embedded HLA (h) and peptide (p) sequences as fixed-length vectors $E_{h}$ and $E_{p}$, respectively. They are processed through dense layers to produce feature-enhanced representations ($E_{h}$ and $E_{p}$), which are merged into a joint vector $E_{h,\;p}$. This joint representation yields an intermediate score (s). Separately, nine IEDB-based predictors generate a score vector $S_{h,\;p}$ for the same pair. The final prediction score y ∈ {0, 1} is made by combining s and $S_{h,\;p}$. Each layer block indicates the number of units (U), activation function (A), and Dropout rate (D), as shown in the legend.

Here, we propose *SiaScoreNet*, a novel pipeline designed to address the HLA-peptide interaction problem. Given an HLA sequence (h) and a peptide sequence (p), the pipeline performs the following main steps (see Fig. 2):

1. Input embedding: The HLA and peptide sequences, h and p, are transformed into their respective encoding vectors, $E_{h}$ and $E_{p}$.
2. IEDB scores: For each HLA-peptide pair <h,p>, a vector $S_{h,p}$ of length 9 is generated, where each element represents the predicted binding score from one of nine IEDB predictors.
3. A three-branch heterogeneous SNN model: The three vectors, $E_{h}$, $E_{p},\;$and $S_{h,p}$, are fed into a three-branch heterogeneous SNN, named *TriSiamHP*, to predict HLA-peptide interaction as a binary outcome y∈{0,1}.

Further details for each step are given below.

#### 2.3.1 Input embedding

In traditional methods for the HLA-peptide interaction problem, input sequences (HLA and peptide sequences) are represented using techniques such as one-hot encoding or substitution matrices like BLOSUM (Henikoff and Henikoff 1992; Chen *et al.* 2021; Ye *et al.* 2021). While somewhat effective, these methods have limitations: one-hot encoding treats amino acids as independent entities, ignoring their biochemical similarities, whereas BLOSUM matrices apply fixed substitution scores that cannot adapt to the specific context of a given dataset.

In contrast, word embedding models like Word2Vec (Mikolov *et al.* 2013), generate low-dimensional representations of sequence elements by learning from their co-occurrence patterns within a dataset. These embeddings capture meaningful properties like physicochemical properties, conserved sequence motifs, and binding-related structural characteristics (Adjuik and Ananey-Obiri 2022).

More recently, protein language models offer a powerful alternative for understanding HLA and peptide sequences. These models encode sequences into continuous vector spaces that reflect structural, functional, and contextual relationships (Song *et al.* 2021; Oviya *et al.* 2024). Pretrained models, such as ESM (Lin *et al.* 2023), were trained on large-scale protein databases to produce context-aware representations of amino acids and sequence fragments, capturing nuanced biological information (Behjati *et al.* 2022).

These models uncover subtle, functionally relevant HLA-peptide relationships often missed by traditional methods. In the following, we evaluate and compare different sequence representations—Word2Vec and pretrained ESM—to identify which provides more informative input representation for predicting HLA–peptide interactions:

- **Representation by Word2Vec:** We employ the Word2Vec approach, treating 3-mers as words and biological sequences as sentences; specifically, we use the skip-gram model, which predicts surrounding 3-mers based on a given 3-mer to capture contextual relationships within the sequence (Bayati *et al.* 2020; Nabeel Asim *et al.* 2020; Ren *et al.* 2022). For example, the sentence ['SDK', 'DKY', 'KYG', 'YGL', 'GLG', 'LGY'] is generated from the peptide sequence “SDKYGLGY” using a 3-mer approach. In our model, each 3-mer is encoded into a 100-dimensional vector using Word2Vec. The final representation of a peptide p and an HLA sequence h are obtained by averaging the vectors of their constituent 3-mers, resulting in fixed-size embeddings $E_{p}\;$and $E_{h}$, with a dimension of 100.
- **Representation by pretrained ESM:** To transform HLA and peptide sequences into vector representations, we use the pretrained ESM2 protein language model (Lin *et al.* 2023). This model, trained on large protein datasets such as UniRef (Suzek *et al.* 2007), excels at capturing and encoding the latent biological information within sequences to study HLA and peptide interactions. By utilizing its deep contextual embeddings, we gain a comprehensive understanding of sequence-specific characteristics, including structural, functional, and interaction-related properties (Rives *et al.* 2021; Totaro *et al.* 2024). These insights are invaluable for critical applications, such as improving the accuracy of HLA-peptide binding predictions. For each peptide p and HLA h, the model generates two 320-dimensional vectors, noted as$\;E_{p}$ and $E_{h}$, respectively. Specifically, for each sequence of length n, we extract a n×320 matrix from the 6^th^ layer of ESM2 model. This layer is selected based on prior observations that intermediate layers in protein language models (Behjati *et al.* 2022) often capture the most informative representation, we compute the average of the embeddings across the sequence length, resulting in a single 320-dimentional vector for each sequence.

#### 2.3.2 IEDB scores

IEDB (Dhanda *et al.* 2019) provides a collection of nine models (Peters and Sette 2005; Moutaftsi *et al.* 2006; Lundegaard *et al.* 2008; Zhang *et al.* 2009; Kim *et al.* 2009; Karosiene *et al.* 2012; Rasmussen *et al.* 2016; Jurtz *et al.* 2017) that use different representations for HLAs and peptides to predict HLA-peptide interactions. While these models focus on distinct aspects of the interaction, they typically lack a unified representation, limiting their ability to fully understand the complexities of the interaction. In contrast, our pipeline, *SiaScoreNet*, integrates the predicted scores from these IEDB models as a third input vector denoted $S_{h,p}$, where ${|S}_{h,p}|=9$. This vector is used alongside the input embeddings of the HLA h and peptide p sequences. By using the complementary strengths of all three inputs for pair <h,p>—HLA embedding ($E_{h}$), peptide embedding ($E_{p}$) and IEDB model outputs ($S_{h,p}$)—we construct a more comprehensive and robust pipeline for predicting HLA-peptide interactions. The nine IEDB-based predictors integrated into $S_{h,p}$ are: NetMHCpan-EL, NetMHCpan-BA, Consensus, ANN (NetMHC), SMMPMBEC, SMM, PickPocket, NetMHCcons, and NetMHCStabPan.

#### 2.3.3 A three-branch heterogeneous SNN model

In the last step of our pipeline, we design a three-branch heterogeneous SNN architecture, named *TriSiamHP*. This model processes three inputs**—**HLA embedding $E_{h},$ peptide embedding $E_{p}$ and IEDB-derived score vector $S_{h,p}$**—**through separate branches as follows:

1. The HLA embedding $E_{h}$ extracted using a model M∈{ESM, Word2Vec}, is passed through a nonlinear function $f_{M}(E_{h}):\;R^{d}\;\to R^{t}$, serving as the first branch of *TriSiamHP* to generate $f_{M}(E_{h})=E_{h}$
2. Similarly, the peptide embedding $E_{p}$, extracted using the same model M, is processed through another nonlinear function $g_{M}(E_{p}):\;R^{d}\;\to R^{t'}$, forming the second branch and producing $g_{M}(E_{p})=E_{p}$.
3. For each HLA-peptide pair <h, p>, a 9-dimensional score vector $S_{h,\;p},\;$is obtained from IEDB predictors and processed through the third branch.

The input, hidden, layers, and latent space of the first and second branches, $f_{M}$ and $g_{M}$, are structurally identical but do not share parameters. The architecture is defined based on the choice of embedding representation M∈{ESM, Word2Vec}:

- For M=Word2Vec:
  - Input dimension: $|E_{h}|=100$ and $|E_{p}|=100$,
  - Hidden layers in $f_{\mathrm{Word}2V\mathrm{ec}}$ and $g_{\mathrm{Word}2V\mathrm{ec}}$: 64→32,
  - Latent space dimension: $|E_{h}|=16$ and $|E_{p}|=16$.
- For M=ESM:
  - Input dimension: $|E_{h}|=320$ and $|E_{p}|=320$,
  - Hidden layers in $f_{\mathrm{ESM}}$ and $g_{\mathrm{ESM}}$: 256→128→64,
  - Latent space dimension: $|E_{h}|=32$ and $|E_{p}|=32$.

This design ensures that while both branches share the same architecture, they learn independent transformations for the HLA and peptide embeddings. For each M∈{ESM, Word2Vec}, the resulting latent representations, $E_{h}$ and $E_{p}$, are combined using one of the following approaches to form a joint representation $E_{h,\;p}$. The following combination methods are selected based on experimentation with various strategies and performance evaluations through trial and error:

-
$E_{h,p}=[E_{h}∥E_{p}]$ : Concatenation of the HLA and peptide embeddings.
-
$E_{h,p}=[\left(E_{h}⊙E_{p})∥|E_{h}-E_{p}\right|]$ : Concatenation of the element-wise product and absolute difference of the embeddings.

We define four versions of *TriSiamHP* model based on the input embedding method M and the latent space combination strategy (see Table 1). These versions do not incorporate the third branch and instead pass the combined latent space through fully connected layers. Specifically, ${\mathrm{TriSiamHP}}_{W}^{C}$ and ${\mathrm{TriSiamHP}}_{W}^{\mathrm{PD}}$ are followed by dense layers of size 32→16→1, while ${\mathrm{TriSiamHP}}_{E}^{C}$ and ${\mathrm{TriSiamHP}}_{E}^{\mathrm{PD}}$ use a deeper architecture with dense layers of size 64→32→16→1.

**Table 1.** Summary of the four TriSiamHP model variants based on the embedding method (Word2Vec or ESM) and the latent space combination strategy (concatenation or element-wise product with absolute difference).

Model version	Embedding method (M)	Latent space combination strategy ($E_{h,p}$)
${\mathrm{TriSiamHP}}_{W}^{C}$	Word2Vec	Concatenation of HLA and peptide embeddings
${\mathrm{TriSiamHP}}_{W}^{\mathrm{PD}}$	Word2Vec	Element-wise product and absolute difference concatenate
${\mathrm{TriSiamHP}}_{E}^{C}$	ESM	Concatenation of HLA and peptide embeddings
${\mathrm{TriSiamHP}}_{E}^{\mathrm{PD}}$	ESM	Element-wise product and absolute difference concatenate

As demonstrated in the Results section, the ESM-based representation is more effective for HLA–peptide prediction. Therefore, we use ${\mathrm{TriSiamHP}}_{E}^{C}$ and ${\mathrm{TriSiamHP}}_{E}^{\mathrm{PD}}$ as the foundation for integrating the third branch.

Building on the latent space combination strategy used in $f_{M}$ and $g_{M}$, we define two extended versions$,\;{\mathrm{TriSiamHP}}_{E,I}^{C}$ and ${\mathrm{TriSiamHP}}_{E,I}^{\mathrm{PD}}$. In both versions, the latent space $E_{h,p}$, with dimensionality ${|E}_{h,p}|=128$, is passed through a deeper architecture composed of dense layers with sizes 64→32→16→8→1. The final single latent vector s is then concatenated with IEDB score vector $S_{h,p}$ (9 dimensions), resulting in a 10-dimentional vector $\left[S∥S_{h,p}\right]$. This combined vector is passed to a single output neuron to generate as output prediction.

All model variants apply Batch Normalization and Dropout (rate = 0.2) after each layer. The models are trained using a batch sized of 256 and a learning rate of 10^-^³, which is dynamically adjusted by a ReduceLROnPlateau scheduler based on validation performance. The Swish activation function is used throughout the network, except for the final output neuron, which uses a sigmoid function. The number of training epochs is determined by monitoring the validation loss.

## 3 Results

In this section, we first examine the individual and combined contributions of our embedding strategies in *SiaScoreNet*, comparing pretrained ESM embeddings against Word2Vec 3-mer representations. We then assess the benefit of integrating IEDB-derived binding scores into our pipeline before benchmarking *SiaScoreNet* against the state-of-the-art HLA–peptide predictors. To ensure a fair evaluation, we introduce a peptide similarity filtering step to control for potential data leakage between training and test sets. Next, we explore allele-specific discrimination and early-retrieval performance across five common HLA subtypes and extend this analysis to entirely unseen HLA alleles to evaluate generalization. Finally, we present a focused case study on HPV16 E6/E7–derived peptides to demonstrate the real-world applicability of *SiaScoreNet* in therapeutic epitope discovery.

To provide a multifaceted view of model performance, we employ a comprehensive suite of evaluation metrics. Threshold-dependent metrics—including Accuracy (ACC), Precision, Recall, Specificity, F1-score, and the Matthews Correlation Coefficient (MCC)—are used to assess classification efficacy at a standard decision boundary. In parallel, we use threshold-independent metrics to evaluate the models’ ability to rank true binders over non-binders. These include the Area Under the Receiver Operating Characteristic Curve (AUC), the Area Under the Precision-Recall Curve (AUPR), and the partial AUC at a False Positive Rate of 0.1 (AUC 0.1), which specifically measures performance in the critical early retrieval phase. In particular, we include AUC 0.1 alongside the overall AUC, as it highlights the portion of the ROC curve where the false positive rate (FPR) is low—an area that is especially important in high-precision applications.

### 3.1 Performance impact of ESM and Word2Vec embeddings on SiaScoreNet

In this subsection, we evaluate the effectiveness of ESM and Word2Vec embeddings in capturing the latent characteristics of HLA and peptide sequences within the TriSiamHP model, which forms the third step of the *SiaScoreNet* pipeline. By comparing these two embedding methods, our goal is to determine which provides more informative and discriminative representations for improving HLA–peptide interaction prediction accuracy.

The evaluation is performed on dataset $D_{1}$ using 5-fold cross-validation. In each fold, one subset is used as the test set, while the remaining four are split into 90% for training and 10% for validation.

We evaluate four versions of the *TriSiamHP* model listed in Table 2, using only the two main branches of the SNN, providing peptide and HLA embeddings as inputs, excluding the third input branch. Based on the average performance across all folds and multiple evaluation metrics (Table 2), ESM embeddings are found to be the most effective. This is because they are generated by a pretrained large language model that captures representations based on full sequences. Consequently, the models ${\mathrm{TriSiamHP}}_{E}^{C}$ and ${\mathrm{TriSiamHP}}_{E}^{\mathrm{PD}}$are selected for further assessment.

**Table 2.** Average performance of the four *TriSiamHP* model variants (excluding the third branch) across 5-fold cross-validation on dataset D1.

Metric	${\mathrm{TriSiamHP}}_{W}^{C}$	${\mathrm{TriSiamHP}}_{W}^{\mathrm{PD}}$	${\mathrm{TriSiamHP}}_{E}^{C}$	${\mathrm{TriSiamHP}}_{E}^{\mathrm{PD}}$
AUC	0.7878 ± 0.0033	0.8029 ± 0.0046	0.9561 ± 0.0010	**0.9591 ± 0.0011**
AUPR	0.7765 ± 0.0042	0.7895 ± 0.0085	0.9522 ± 0.0017	**0.9559 ± 0.0015**
MCC	0.4248 ± 0.0092	0.4532 ± 0.0077	0.7866 ± 0.0027	**0.7955 ± 0.0024**
ACC	0.7119 ± 0.0045	0.7255 ± 0.0042	0.8931 ± 0.0014	**0.8977 ± 0.0011**
Recall	0.7490 ± 0.0088	0.7747 ± 0.0080	**0.9094 ± 0.0033**	0.9092 ± 0.0060
Precision	0.6981 ± 0.0043	0.7063 ± 0.0076	0.8811 ± 0.0051	**0.8891 ± 0.0019**
F1-score	0.7226 ± 0.0055	0.7388 ± 0.0050	0.8950 ± 0.0018	**0.8990 ± 0.0020**
Specificity	0.6745 ± 0.0036	0.6762 ± 0.0102	0.8768 ± 0.0048	**0.8860 ± 0.0043**

### 3.2 Incorporating IEDB scores into SiaScoreNet

Here, we evaluate whether integrating the score prediction of nine IEDB predictors as a feature vector in the third branch of the SNN, alongside ESM-embedded HLA and peptide sequences in the first and second branches, can improve the predictive performance of *TrsiSiamHP* within the *SiaScoreNet* pipeline. This evaluation focuses on the extended models ${\mathrm{TriSiamHP}}_{E,I}^{C}$ and ${\mathrm{TriSiamHP}}_{E,I}^{\mathrm{PD}},$ and is conducted using 5-fold cross-validation on dataset $D_{1}$. The performance of the extended *TriSiamHP* models is reported in the first and second rows of Table 3. The first and second rows present two model variants, ${\mathrm{TriSiamHP}}_{E,I}^{C}$ and ${\mathrm{TriSiamHP}}_{E,I}^{\mathrm{PD}}$. The main difference between these variants lies in how the embeddings extracted from the HLA and peptide branches are integrated. These integrated embeddings are then combined with the nine prediction scores from the IEDB models.

**Table 3.** Average 5-fold cross-validation *performance of extended TriSiamHP models*, incorporating IEDB Score predictions as the third-branch feature vector on Dataset $D_{1}$.

Metric	${\mathrm{TriSiamHP}}_{E,I}^{C}$	${\mathrm{TriSiamHP}}_{E,I}^{\mathrm{PD}}\;$	${\mathrm{TriSiamHP}}_{E,I}^{\mathrm{PD}}$ in absence of Consensus and NetMHCcons models
AUC	0.9764 ± 0.0008	**0.9769 ± 0.0005**	0.9766 ± 0.0009
AUPR	0.9766 ± 0.0010	**0.9769 ± 0.0010**	0.9767 ± 0.0011
MCC	0.8541 ± 0.0041	**0.8556 ± 0.0031**	0.8529 ± 0.0046
ACC	0.9270 ± 0.0020	**0.9278 ± 0.0016**	0.9264 ± 0.0023
Recall	0.9297 ± 0.0025	**0.9314 ± 0.0023**	0.9280 ± 0.0033
Precision	0.9251 ± 0.0034	0.9251 ± 0.0030	**0.9254 ± 0.0029**
F1-score	0.9274 ± 0.0022	**0.9282 ± 0.0016**	0.9267 ± 0.0025
Specificity	0.9243 ± 0.0034	**0.9769 ± 0.0005**	0.9248 ± 0.0034

A comparison between ${\mathrm{TriSiamHP}}_{E}^{C}$ (Table 2) and ${\mathrm{TriSiamHP}}_{E,I}^{C}$ (Table 3) indicates that incorporating the predictor scoring vector enhances the performance of HLA-peptide interaction prediction. A similar improvement is observed when comparing ${\mathrm{TriSiamHP}}_{E}^{\mathrm{PD}}$ (Table 2) and ${\mathrm{TriSiamHP}}_{E,I}^{\mathrm{PD}}$ (Table 3).

Overall, the average performance across the 5-fold cross-validation (Table 3) shows that ${\mathrm{TriSiamHP}}_{E,I}^{\mathrm{PD}}$ outperforms ${\mathrm{TriSiamHP}}_{E,I}^{C}$. The training, validation, and test loss curves for each epoch and fold of ${\mathrm{TriSiamHP}}_{E,I}^{\mathrm{PD}}$ on the $D_{1}$ dataset is provided in Fig. S1.

It is important to note that some of the IEDB predictors are consensus models—such as Consensus (Moutaftsi *et al.* 2006) and NetMHCcons (Karosiene *et al.* 2012)—which are constructed by aggregating the outputs of other individual predictors within IEDB. To find the importance of these consensus models, we exclude the scores of these ensemble predictors, reducing the length of the scoring vector $S_{h,p}$ from 9 to 7. The third row of Table 3 presents the average results across 5-fold cross-validation, indicating that the consensus predictor scores remain contributors to the model’s performance when used as input features.

In the following, we assess that the SNN architecture significantly enhances HLA-peptide interaction prediction, whereas baseline models fail to achieve comparable performance, even when provided with ESM embedding of HLA and peptide sequences along with the nine IEDB predictor scores as input features. Table S3 presents the results of these baseline models under 5-fold cross-validation on dataset $D_{1}$. Among them, the random forest classifier (RFC) shows performance closet to that of ${\mathrm{TriSiamHP}}_{E,I}^{\mathrm{PD}}$.

### 3.3 Comparison of SiaScoreNet pipeline with the state-of-art predictors

**Figure 3.**
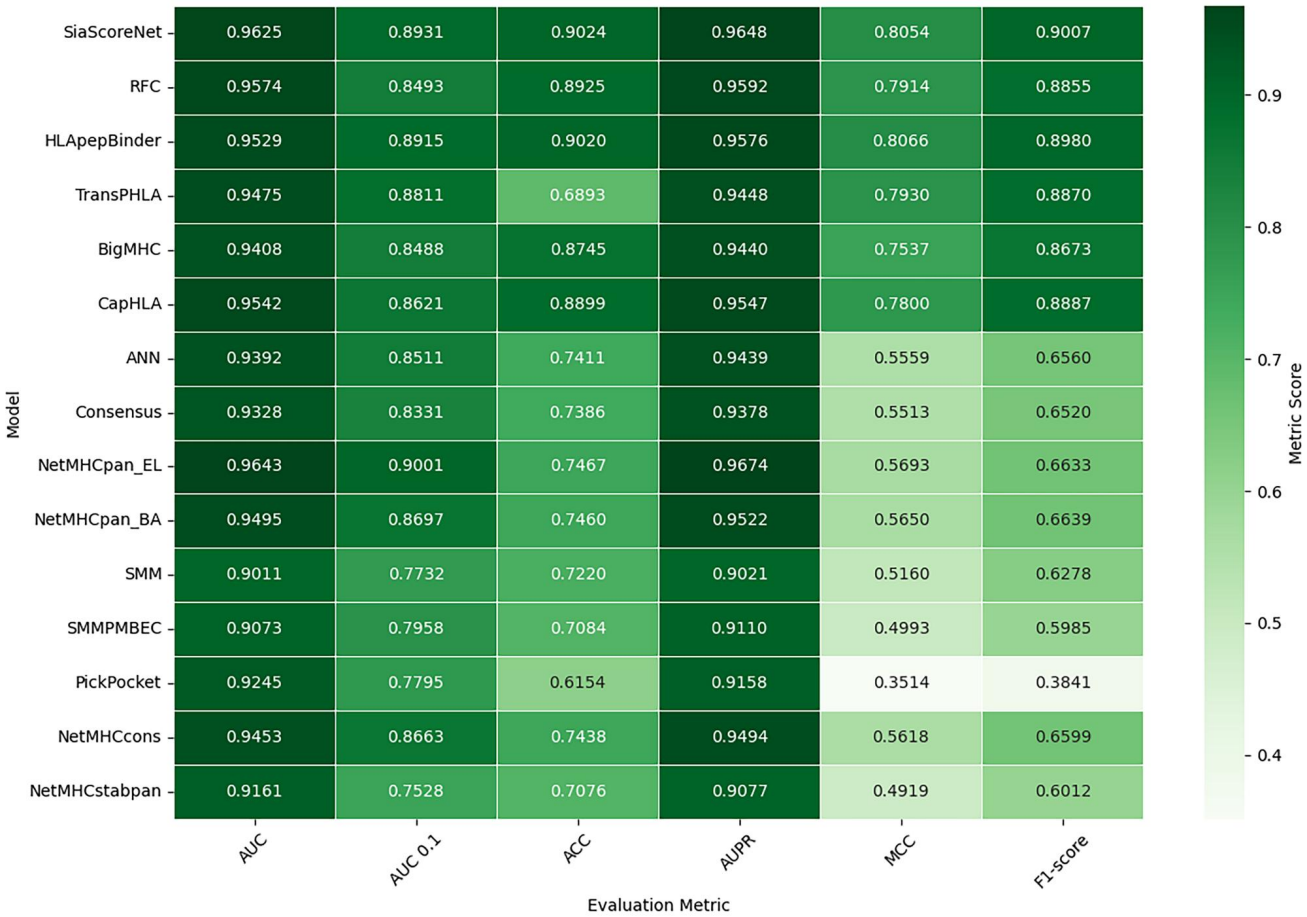
Performance comparison of *SiaScoreNet* with the state-of-art predictors for HLA–peptide interaction prediction.

The *TriSiamHP* model (specifically, the ${\mathrm{TriSiamHP}}_{E,I}^{\mathrm{PD}}$ variant), used as the third step of *SiaScoreNet* pipeline, is trained on dataset $D_{1}$ and evaluated on dataset $D_{2}$. We compare its performance against 14 existing HLA-peptide interaction prediction models. Figure 3 presents a comparative evaluation of *SiaScoreNet* and several state-of-the-art models for HLA–peptide interaction prediction using threshold-independent metrics, including AUC, AUC 0.1, and AUPR. *SiaScoreNet* achieves consistently high scores across these metrics, with an AUC of 0.9625, an AUC 0.1 of 0.8931, and an AUPR of 0.9648, placing it among the top-performing models in all categories. In particular, its AUPR and AUC values reflect a strong ability to distinguish between binders and non-binders, even under class imbalance conditions. While models like NetMHCpan_EL (Reynisson *et al.* 2020) and HLApepBinder (Saadat *et al.* 2024) also show competitive results in some metrics, *SiaScoreNet* demonstrates more consistent and robust performance overall. These findings highlight *SiaScoreNet’*s strong discriminative capability without relying on threshold-based evaluations.

### 3.4 Controlling data leakage through peptide similarity filtering

**Figure 4.**
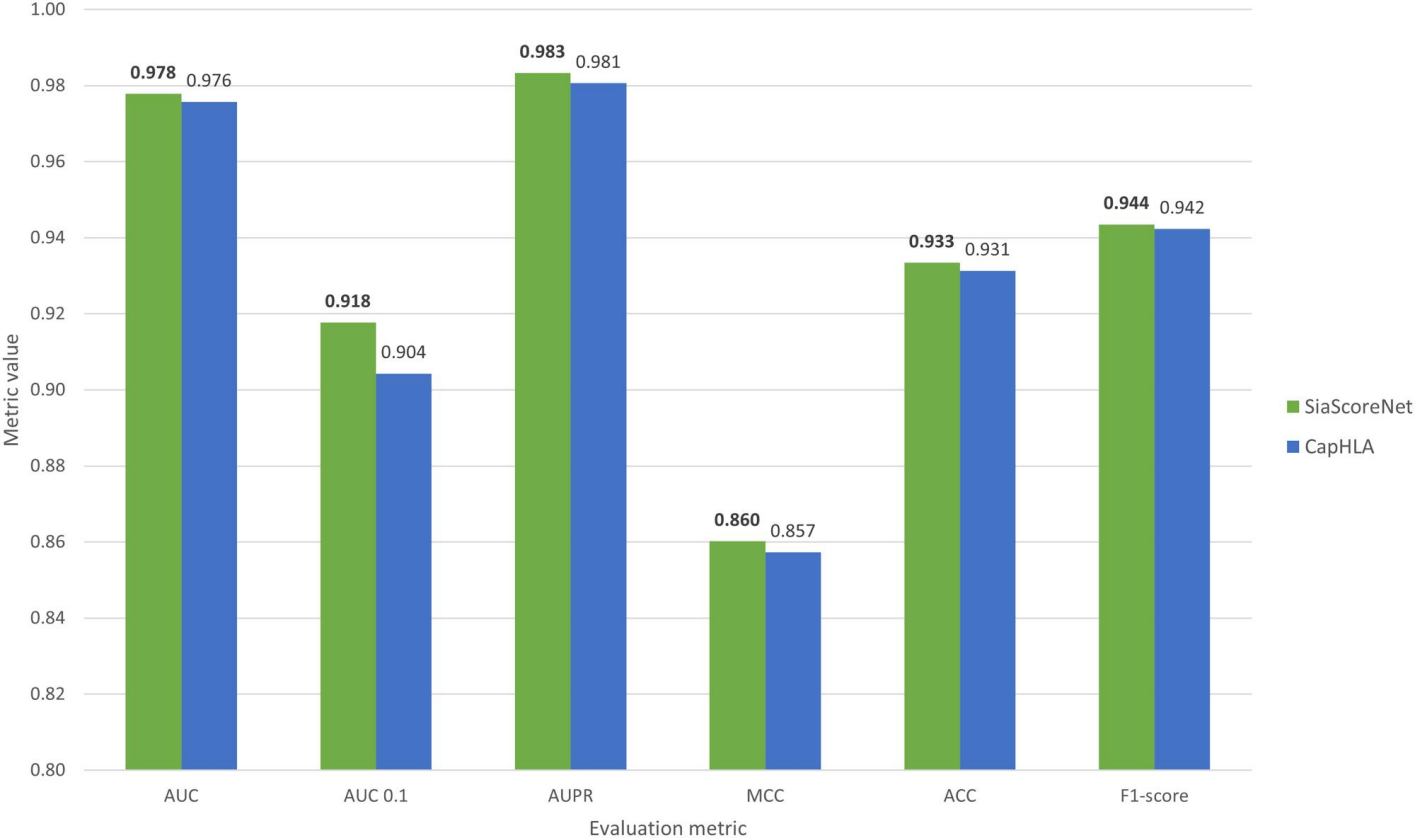
Performance comparison between *SiaScoreNet* and CapHLA on a test set of ten randomly selected peptides and their similar peptides—excluded from D_1_—with both models trained on the remaining D_1_ data. The y-axis starts at 0.8.

An important challenge arises from the overlapping binding preferences of many HLAs, which can obscure a clear evaluation of how the model generalizes to entirely new peptides, particularly when no similar sequences are present in the training data. As a result, when the same peptide appears in both the training and test sets—paired with different HLAs—the model may unintentionally rely on information seen during training to make predictions in the test set, which can lead to an overestimation of its true generalization ability. To mitigate this, we randomly select 10 peptides from dataset $D_{1}$ and designate all peptides in $D_{1}$ that share more than 50% similarity (based on the BLOSUM62 substitution matrix) with any of these 10 peptides as the test set. The remaining HLA-peptide pairs are used as the training set. This procedure yields in 32329 HLA–peptide pairs in the test set and 91759 in the training set. We train our model, as well as the recently proposed CapHLA (Chang and Wu 2024) model, on this training set and evaluate both models on the constructed test set. Figure 4 shows that our model outperforms CapHLA across all evaluation metrics, even when similar peptides are excluded from the training set, demonstrating its superior generalization capability.

**Figure 5.**
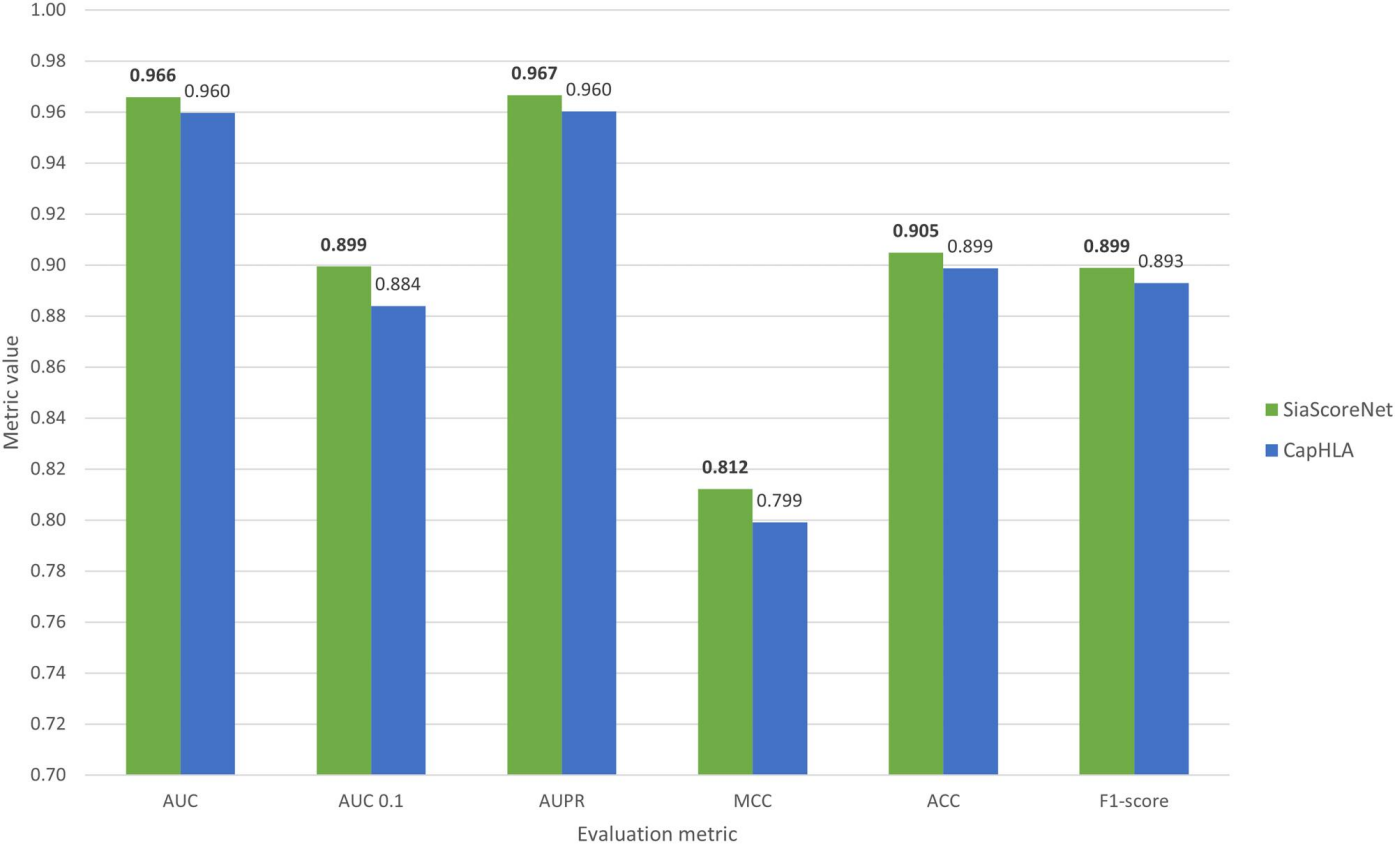
Performance comparison between *SiaScoreNet* and CapHLA on non-overlapping peptide sets. The y-axis starts at 0.7.

For further analysis, we have removed all peptides from dataset $D_{2}$ that are also present in dataset $D_{1}$ to ensure a strict separation and prevent any potential data leakage at the peptide level. In total, 2822 overlapping peptides are removed. The updated results, presented in Fig. 5, highlight the improved generalization ability of our model compared to CapHLA under this more rigorous evaluation protocol.

### 3.5 Allele-specific performance and low-FPR discrimination

**Figure 6.**
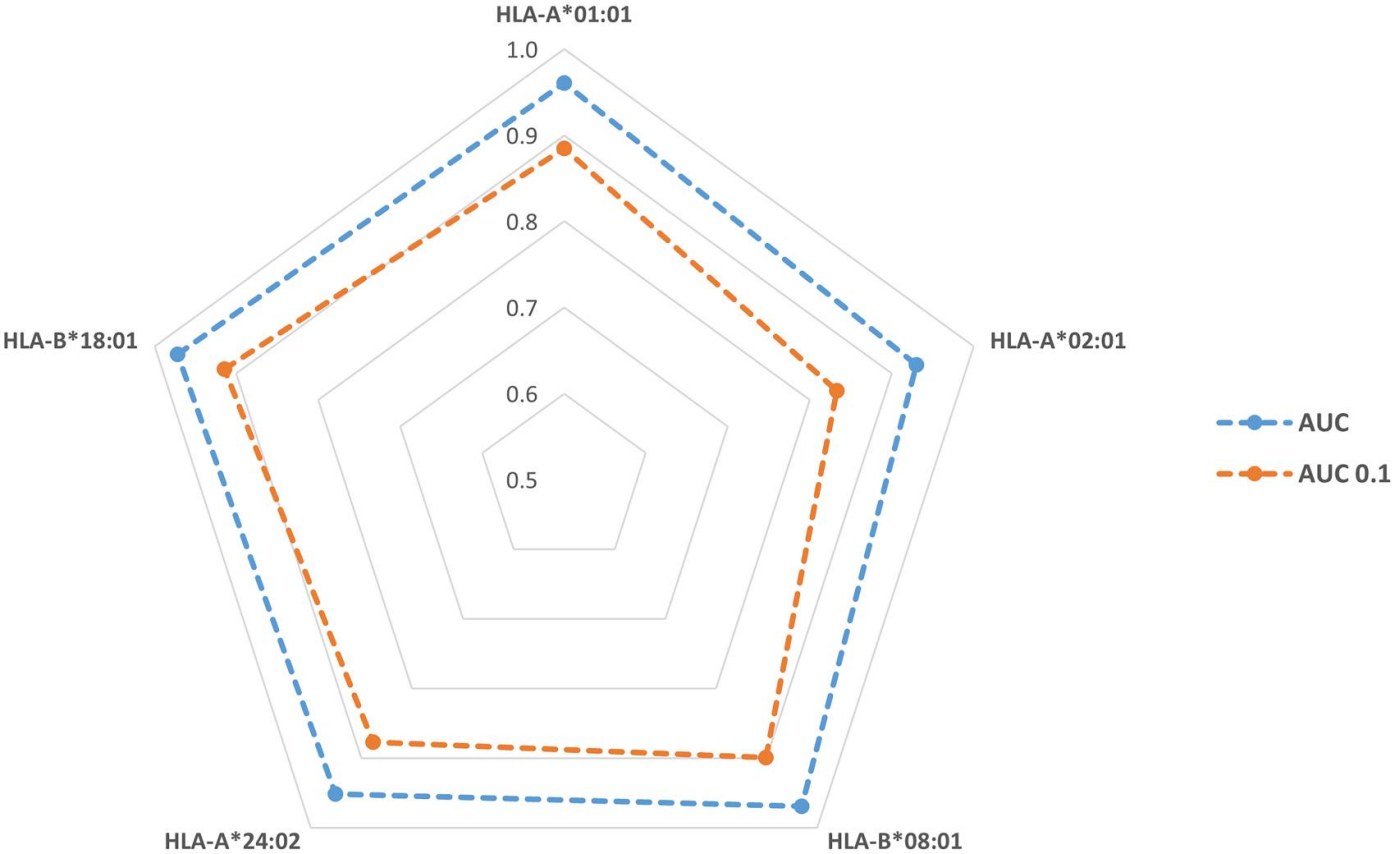
Performance of *SiaScoreNet* on five HLA subtypes in dataset $D_{2}$, evaluated by AUC and AUC 0.1.

In this subsection, our primary goal is to demonstrate that our pipeline is not biased toward specific HLA subtypes. To this end, we train the model on dataset $D_{1}$ and evaluate its performance separately on five distinct HLA subtypes from dataset $D_{2}$. Figure 6 reports the AUC scores for these subtypes. Across the five HLA subtypes (Fig. 6), *SiaScoreNet* demonstrates consistently strong discrimination and early‐retrieval performance:

-
AUC ranges from **0.9304** to **0.9719** (mean ≈ 0.9567), indicating excellent separation of binders and non-binders across all thresholds.
-
AUC 0.1 ranges from **0.8332** to **0.9146** (mean ≈ 0.8816), showing that even when limiting the false positive rate to 10%, the model still prioritizes true binders very effectively.
- The average gap between AUC and AUC 0.1is about **0.075**, a typical drop when focusing on the most stringent decision region, yet the AUC 0.1values remain high.

### 3.6 Evaluating model performance on unseen HLA subtypes

We aim to investigate how the model performs when it has not seen a particular HLA subtype during training. In dataset $D_{1}$, there are 73 HLA subtypes. We exclude each HLA subtype one at a time, train our model on the remaining data, and then test it on the excluded HLA subtype The average and standard deviation of ACC, AUC, and F1-score are 0.9235 ± 0.001992, 0.9745 ± 0.000519, and 0.9217 ± 0.002795, respectively. These results demonstrate that the model generalizes well and is not overly dependent on specific HLA subtypes. Notably, despite the varying number of samples per subtype, the performance metrics remain consistent across all experiments, indicating low variance. Detailed results are presented in Fig. S2.

### 3.7 Case study: HPV16 E6/E7

HPV is one of the most prevalent sexually transmitted infections worldwide and is known to cause several types of cancers, including cervical cancer. While preventive HPV vaccines exist, their therapeutic efficacy remains limited, highlighting the need for accurate identification of immunogenic epitopes to guide vaccine development and immunotherapy strategies (Skeate *et al.* 2016).

To assess the ability of different models to predict HLA-peptide binding relevant to HPV antigens, we select a subset of 212 experimentally verified HLA- peptide binding samples derived from the E6 and E7 proteins of HPV16. These peptides are considered binders based on IC50 ≤ 500 nM, which aligns with commonly used thresholds in HLA-peptide prediction tasks (Xiong *et al.* 2022).

**Figure 7.**
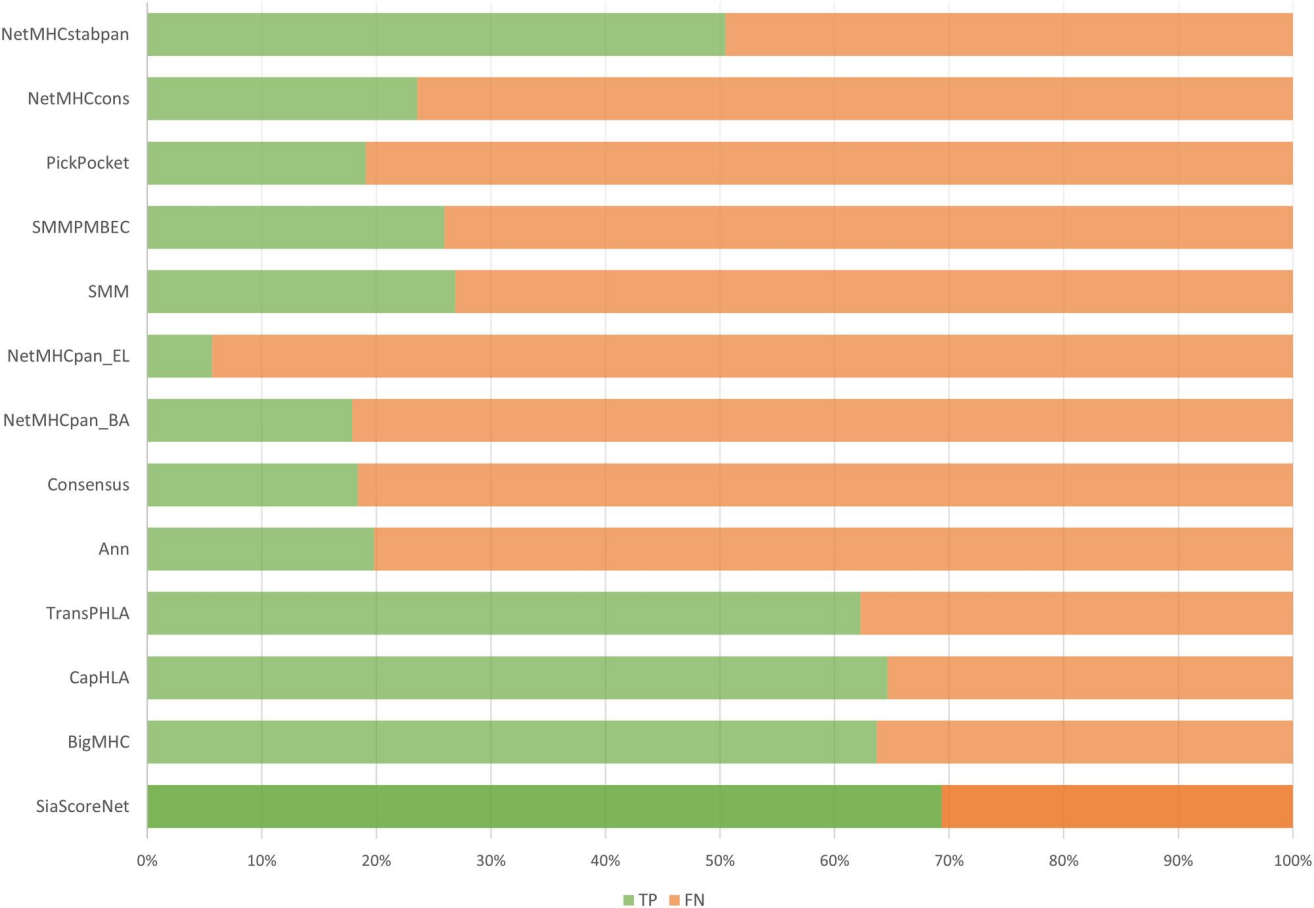
Comparison of *SiaScoreNet* with 12 state-of-the-art predictors in identifying HPV-derived HLA-peptide binders. The horizontal stacked bars display the proportion of true positives (TP, green) and false negatives (FN, orange) for each model on a benchmark set of 212 experimentally verified binders derived from the E6 and E7 proteins of HPV16 (IC50 ≤ 500 nM). *SiaScoreNet* achieves the highest number of true positives (147) and the lowest number of false negatives (65), demonstrating superior recall performance in identifying clinically relevant epitopes for therapeutic vaccine development.

We evaluate our model, *SiaScoreNet*, alongside 12 state-of-the-art methods. As shown in Fig. 7, *SiaScoreNet* achieves the highest number of true positives (147) and the lowest number of false negatives (65) among all models. CapHLA (Chang and Wu 2024) and BigMHC (Albert *et al.* 2023) follows with TP/FN values of 137/75 and 135/77, respectively. In contrast, traditional predictors such as NetMHCpan_EL (Reynisson *et al.* 2020) and PickPocket (Zhang *et al.* 2009) demonstrate lower sensitivity, likely due to their emphasis on precision over recall. More recent models, including *SiaScoreNet*, aim to improve recall—even at the cost of some precision—to better capture a broader range of true binders, which is especially important in therapeutic epitope discovery.

As our study focused exclusively on HLA–peptide pairs specific to HPV16, computing threshold-independent metrics such as AUC and AUC 0.1 posed challenges due to the limited number of experimentally verified binders. To address this, we generate a corresponding set of negative peptides for each positive HLA–peptide binder. Specifically, for every unique positive binder in our dataset, we extract all possible overlapping *n*-mers (where *n* equals the length of the positive peptide) from the HPV16 E6/E7 source proteins. This approach yielded an imbalanced dataset containing both positive and negative examples, thereby allowing a more comprehensive evaluation using both threshold-independent and threshold-dependent metrics.

The comparative evaluation of *SiaScoreNet* against all 12 state-of-the-art predictors is presented in Table 4. In terms of threshold-independent metrics, *SiaScoreNet* demonstrates highly competitive performance. Specifically, while NetMHCcons (0.9549) and Consensus (0.9515) achieve slightly higher AUC values than *SiaScoreNet* (0.9512), all three methods exhibit strong overall discriminative ability. For AUC 0.1, NetMHCcons (0.8775) and NetMHCpan_BA (0.8474) perform best, while *SiaScoreNet* shows a moderate score of 0.8335. Similarly, NetMHCcons (0.6252) and Consensus (0.5783) achieve the highest AUPR values, but *SiaScoreNet* (0.4970) clearly surpasses other deep learning such as BigMHC (0.3597) and CapHLA (0.1630). However, threshold-independent metrics alone cannot fully capture practical utility, as they reflect only the ranking ability across all possible cutoffs without addressing performance at applicable decision thresholds. When focusing on threshold-dependent evaluation, a clearer advantage for *SiaScoreNet* emerges. The F1-score, which quantifies the balance of precision and recall at a fixed cutoff, is the highest for *SiaScoreNet* (0.5213), clearly above NetMHCcons (0.3564) and Consensus (0.3023). This indicates that although consensus-based models achieve excellent AUC, they are less effective at practical threshold-based classification, while *SiaScoreNet* provides better calibration and thus more dependable predictions.

**Table 4.** Performance comparison of *SiaScoreNet* and state-of-the-art predictors on HPV dataset.

Model	AUC	AUC 0.1	AUPR	F1-score
SiaScoreNet	0.9512	0.8335	0.4970	0.5213
BigMHC	0.9158	0.7606	0.3597	0.4293
CapHLA	0.9165	0.7768	0.1630	0.4295
TransPHLA	0.9178	0.8078	0.4527	0.4944
Ann	0.9469	0.8380	0.5559	0.3206
Consensus	0.9515	0.8380	0.5783	0.3023
NetMHCpan_BA	0.9492	0.8474	0.5769	0.2934
NetMHCpan_EL	0.9137	0.7835	0.4516	0.1067
SMM	0.9160	0.7882	0.4416	0.3677
SMMPMBEC	0.9304	0.8105	0.4966	0.3793
PickPocket	0.9277	0.8001	0.4389	0.2701
NetMHCcons	0.9549	0.8775	0.6252	0.3564
NetMHCstabpan	0.9303	0.8101	0.5249	0.5209

To further assess model performance under realistic peptide-specific conditions, we evaluate all 13 predictors using per-positive-peptide test sets. For each peptide, overlapping negative decoys are generated and metrics are computed individually. Table 5 reports the mean and standard deviation of AUC and AUC 0.1 for these peptide-specific sets for all predictors. While NetMHCcons attains the highest mean AUC (0.9609 ± 0.1009) and AUC 0.1 (0.8798 ± 0.1718), *SiaScoreNet* closely follows with values of 0.9594 ± 0.0957 (AUC) and 0.8683 ± 0.1668 (AUC 0.1).

**Table 5.** Performance comparison of *SiaScoreNet* and state-of-the-art predictors on HPV peptide-specific test sets.

Method	AUC 0.1		AUC	
	Mean	Std	Mean	Std
Ann	0.8677	0.1767	0.9577	0.1055
BigMHC	0.7719	0.2130	0.9158	0.1330
CapHLA	0.8021	0.2079	0.9203	0.1635
Consensus	0.8666	0.1782	0.9589	0.0918
NetMHCcons	0.8798	0.1718	0.9609	0.1009
NetMHCpan_BA	0.8756	0.1772	0.9579	0.1134
NetMHCpan_EL	0.8225	0.2014	0.9390	0.1263
NetMHCstabpan	0.8331	0.2060	0.9404	0.1213
PickPocket	0.8370	0.1900	0.9403	0.1289
SiaScoreNet	0.8683	0.1668	0.9594	0.0957
SMM	0.7980	0.2173	0.9045	0.1693
SMMPMBEC	0.8165	0.2119	0.9263	0.1404
TransPHLA	0.8436	0.1986	0.9283	0.1538

Overall, in the HPV case study, *SiaScoreNet* demonstrated highly competitive performance by achieving the highest sensitivity (Recall) and F1-score, striking an effective balance between maximizing the identification of true binders and ensuring practical utility.

## 4 Discussions and conclusion

This study presented *SiaScoreNet*, a novel and efficient framework for predicting HLA class I peptide interactions, which plays a pivotal role in vaccine design and personalized immunotherapy. By using ESM and a heterogeneous SNN, *SiaScoreNet* effectively captures complex biological patterns from variable-length protein sequences, offering enhanced prediction performance over traditional fixed-length models. Through rigorous evaluations against 14 state-of-the-art methods and external datasets, *SiaScoreNet* consistently outperformed competitors in key metrics such as precision, recall, and F1-score, while demonstrating superior computational efficiency. Additionally, a case study on HPV-specific epitopes was presented to highlight the practical utility of the model in identifying true binders, which are critical for therapeutic vaccine development. While currently focused on class I HLA molecules, *SiaScoreNet* shows strong potential for expansion into class II predictions, which are increasingly recognized as vital in cancer immunotherapy. Future improvements, including integration of gene expression data, flanking regions, and support for diverse mutation types, will further enhance the model’s applicability in real-world, high-throughput immunopeptidome analysis and precision oncology.

## Supplementary Material

vbaf248_Supplementary_Data

## Data Availability

The data and source code for prediction and experiments presented in this study is publicly available in the *SiaScoreNet* repository hosted on GitHub: https://github.com/CBRC-lab/SiaScoreNet.
