# Supplementary material for: SiaScoreNet: a siamese neural network-based model integrating prediction scores for HLA-peptide interaction prediction
Source: Bioinform Adv. 2025 Nov 19;5(1):vbaf248. doi: 10.1093/bioadv/vbaf248 (PMC12641608; doi:10.1093/bioadv/vbaf248)
Supplement: vbaf248_Supplementary_Data [file vbaf248_supplementary_data.docx]

# Dataset

In our study, we employ the “Independent test dataset” as $D_{1}$ and “External test dataset” as $D_{2}$defined for both positive and negative HLA-peptide interaction data utilized by TransPHLA (1). Specifically:​

- **Positive data:** They obtained HLA-peptide binding data from the Anthem30 dataset (2).
- **Negative data:** They generated negative samples by randomly selecting peptide segments from source proteins within the IEDB HLA immunopeptidomes, ensuring that for each peptide length and HLA subtype, the negative peptides were derived from the same protein sources as the positive samples. This approach aligns with previous studies, as referenced in TransPHLA's methodology. Although this strategy may inadvertently include some false negatives, prior research indicated that the proportion of such peptides was minimal and could be disregarded.​

## Dataset $D_{1}$

The set of peptides and HLAs in dataset $D_{1}$ are shown by $P^{D_{1}}$ and $H^{D_{1}}$, respectively. In addition, the association matrix of this dataset is named $Y_{73\times115953}^{D_{1}}$. We divide dataset $D_{1}$ into two independent sets $D_{1}^{Pos}$ and $D_{1}^{Neg}$ named positive pair and negative pair sets, respectively as follows:

$$D_{1}^{Pos}=\left\{ <h_{i},p_{j}> \right| h_{i}\in H^{D_{1}},p_{j}\in P^{D_{1}}, y_{i,j}^{D_{1}}=1\},$$

$$D_{1}^{Neg}=\{<h_{i},p_{j}>| h_{i}\in H^{D_{1}},p_{j}\in P^{D_{1}}, y_{i,j}^{D_{1}}=0\},$$

where $D_{1}=D_{1}^{Pos}\cup D_{1}^{Neg}$. Further details regarding this dataset can be found in Table S1.

**Table S1.** Details of dataset $D_{1}$. Number of HLAs, peptides, positive pairs, and negative pairs for each HLA group.

| **HLA groups** | $\boldsymbol{n=\vert}\boldsymbol{H}^{\boldsymbol{D}_{\boldsymbol{1}}}\boldsymbol{\vert}$ | $\boldsymbol{m=\vert P}^{\boldsymbol{D}_{\boldsymbol{1}}}$**\|** | $\boldsymbol{\vert}\boldsymbol{D}_{\boldsymbol{1}}^{\boldsymbol{Pos}}\boldsymbol{\vert}$ | $\boldsymbol{\vert}\boldsymbol{D}_{\boldsymbol{1}}^{\boldsymbol{Neg}}\boldsymbol{\vert}$ |
| --- | --- | --- | --- | --- |
| A | 34 | 48306 | 25484 | 25357 |
| B | 29 | 55668 | 28817 | 28670 |
| C | 10 | 14896 | 7799 | 7781 |
| A, B, C | 73 | 115953 | 62100 | 61808 |

## Dataset $D_{2}$

The details of dataset $D_{2}$ is available in Table S2.

**Table S2.** Details of dataset $D_{2}.$ Number of HLAs, peptides, positive pairs, and negative pairs for each HLA group.

| **HLA groups** | $\boldsymbol{n=\vert}\boldsymbol{H}^{\boldsymbol{D}_{\boldsymbol{2}}}\boldsymbol{\vert}$ | $\boldsymbol{m=\vert P}^{\boldsymbol{D}_{\boldsymbol{2}}}$**\|** | $\boldsymbol{\vert}\boldsymbol{D}_{\boldsymbol{2}}^{\boldsymbol{Pos}}\boldsymbol{\vert}$ | $\boldsymbol{\vert}\boldsymbol{D}_{\boldsymbol{2}}^{\boldsymbol{Neg}}\boldsymbol{\vert}$ |
| --- | --- | --- | --- | --- |
| A | 3 | 22324 | 11472 | 11427 |
| B | 2 | 72080 | 36575 | 36516 |
| A, B | 5 | 93154 | 48047 | 47943 |

# Loss curve analysis across for the ${TriSiamHP}_{E,I}^{\mathbf{PD}}$ model on the $D_{1}$ dataset

Here, the training, validation, and test loss curves for each epoch of every fold on the $D_{1}$dataset using the ${TriSiamHP}_{E,I}^{\mathrm{PD}}$ model, the final step of the *SiaScoreNet* pipeline, are presented. All losses are computed using binary cross-entropy loss, which is appropriate for the pairwise classification task addressed by the model.  Figure S1 illustrates these loss curves across 5-fold cross-validation.

The training loss shows a rapid decrease across all folds within the first 5–10 epochs, indicating fast convergence. Following this initial drop, the loss flattens and stabilizes around 0.19–0.20. The similarity of the curves across folds reflects consistent training behavior and stable optimization.

The validation loss generally follows the same downward trend, with convergence typically occurring between epochs 10 and 15. Minor fluctuations are observed, particularly in folds 2 and 3, but all remain within a narrow range (~0.19–0.22), indicating low variance and no evidence of overfitting. Training is governed by early stopping based on validation loss, and the number of training epochs for each fold is automatically determined by this criterion, typically halting training between epochs 12 and 18.

The test loss curves closely mirror the validation trends, further supporting the model’s ability to generalize. After some early fluctuations, the test losses stabilize around 0.19–0.21 across all folds, demonstrating robust and reliable performance on unseen data.

Overall, the ${TriSiamHP}_{E,I}^{\mathrm{PD}}$ model exhibits efficient learning, strong convergence, and excellent generalization across all folds. The minor variations in validation and test losses are well within expected ranges, confirming the model’s stability and effectiveness in cross-validation.


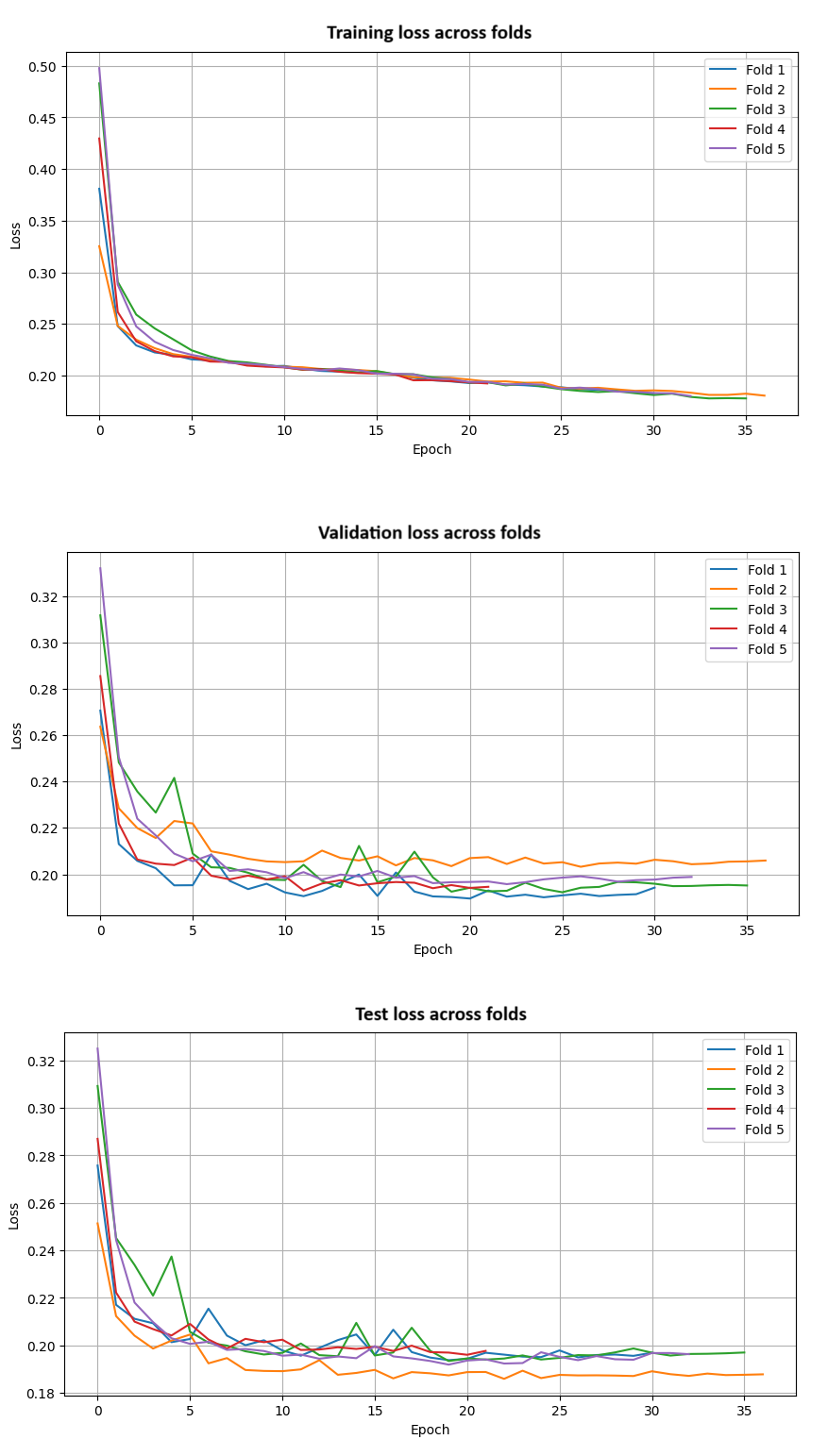


**Figure S1.** Training, validation, and test loss curves across 5-fold cross-validation on the $D_{1}$dataset using the ${TriSiamHP}_{E,I}^{PD}$ model, the final component of the SiaScoreNet pipeline.

# Performance of baseline models using ESM embeddings and IEDB scores for HLA–peptide interaction prediction

We concatenate the peptide embedding, HLA embedding, and IEDB score vector as input features and feed them into baseline models. Table S3 presents the results of these models using 5-fold cross-validation on dataset $D_{1}$.

**Table S3.** Performance of baseline models using ESM embeddings and IEDB score predictions as input features under 5-fold cross-validation on dataset D₁.

| Baseline model | AUC | AUPR | MCC | ACC | Recall | Precision | F1-score | Specificity |
| --- | --- | --- | --- | --- | --- | --- | --- | --- |
| Logistic Regression Classifier | 0.9640**±** 0.0019 | 0.9644**±** 0.0018 | 0.8183**±** 0.0063 | 0.9088**±** 0.0032 | **0.9283± 0.0034** | 0.8939 **±** 0.0048 | 0.9108 **±** 0.0031 | 0.8892 **±.** 0.0053 |
| Random Forest Classifier (RFC) | **0.9754± 0.0006** | **0.9730± 0.0008** | **0.8574± 0.0046** | **0.9287± 0.0023** | 0.9232**±** 0.0035 | **0.9337 ± 0.0027** | **0.9284 ± 0.0025** | **0.9341 ± 0.0028** |
| KNN Classifier | 0.8924**±** 0.0020 | 0.8534**±** 0.0041 | 0.6466**±** 0.0042 | 0.8192**±** 0.0025 | 0.8992**±** 0.0022 | 0.7757 **±** 0.0045 | 0.8329 **±** 0.0031 | 0.7388 **±.** 0.0034 |
| Naive Bayes Classifier | 0.9595**±** 0.0010 | 0.9606**±** 0.0017 | 0.6948**±** 0.0276 | 0.8350**±** 0.0174 | 0.9666**±** 0.0052 | 0.7665 **±** 0.0245 | 0.8547 **±** 0.0133 | 0.7030 **±.** 0.0385 |

## Evaluating model performance on unseen HLA subtypes


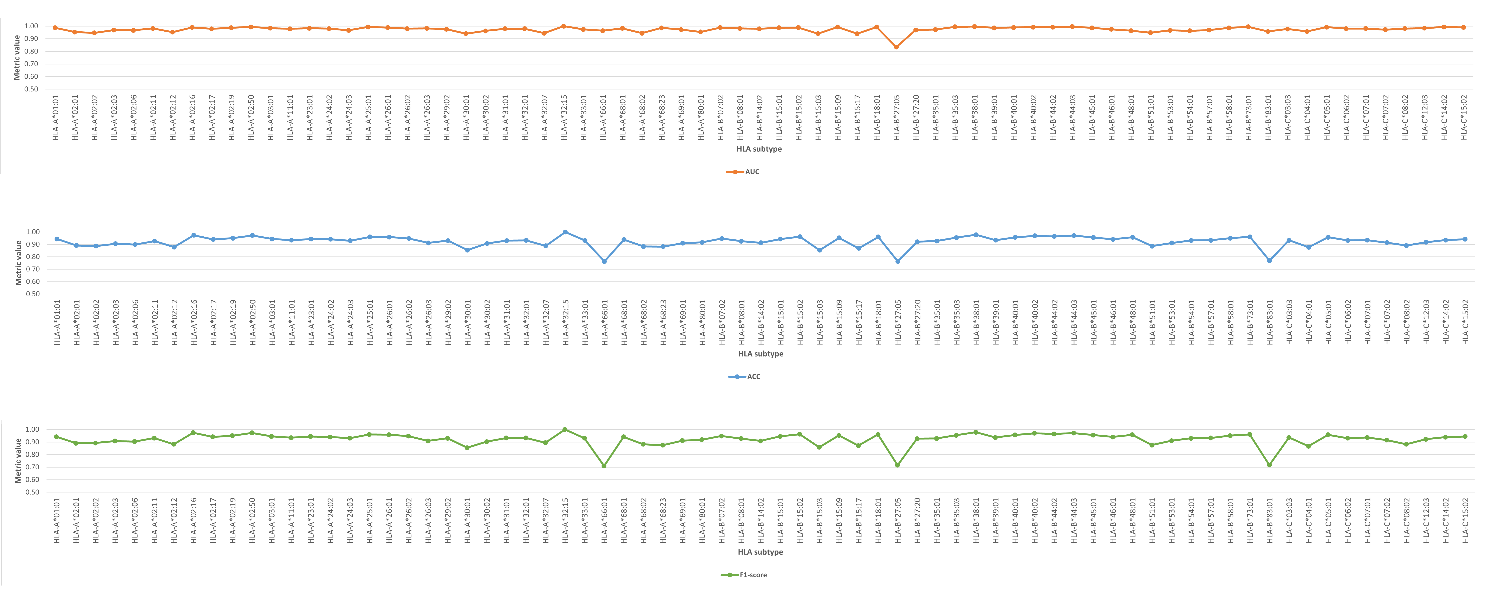


***Figure S2.*** *Leave-One-HLA-Out performance evaluation of SiaScoreNet on dataset D₁. Each HLA subtype on the x-axis is left out from training and used only in test dataset. The vertical axis shows the individual performance metric values — AUC (orange), Accuracy (blue), and F1-score (green).*

# References

1. Chu Y, Zhang Y, Wang Q, Zhang L, Wang X, Wang Y, et al. A transformer-based model to predict peptide–HLA class I binding and optimize mutated peptides for vaccine design. Nat Mach Intell [Internet]. 2022 Mar 23;4(3):300–11. Available from: https://www.nature.com/articles/s42256-022-00459-7

2. Mei S, Li F, Xiang D, Ayala R, Faridi P, Webb GI, et al. Anthem: a user customised tool for fast and accurate prediction of binding between peptides and HLA class I molecules. Brief Bioinform. 2021 Sep 2;22(5).
